# Abrogation of TNFα Production during Cancer Immunotherapy Is Crucial for Suppressing Side Effects Due to the Systemic Expression of IL-12

**DOI:** 10.1371/journal.pone.0090116

**Published:** 2014-02-28

**Authors:** Bibiana Barrios, Natalia S. Baez, Della Reynolds, Pablo Iribarren, Hugo Cejas, Howard A. Young, Maria Cecilia Rodriguez-Galan

**Affiliations:** 1 Inmunología, CIBICI-CONICET, Facultad de Ciencias Químicas, Universidad Nacional de Córdoba, Córdoba, Argentina; 2 Laboratory of Experimental Immunology, Cancer and Inflammation Program, Center for Cancer Research, National Cancer Institute, National Institutes of Health, Frederick, Maryland, United States of America; Virginia Tech University, United States of America

## Abstract

For more than a decade, the cytokine interleukin-12 (IL-12) has been utilized, either alone or in combination with other drugs, as a treatment for cancer. The numerous anti-tumor properties of IL-12 still generate interest in the clinical use of this cytokine, even though it has demonstrated toxicity when administrated systemically. As an approach to overcome this toxicity, numerous laboratories have attempted to induce IL-12 expression at the site of the tumor. However for tumors that are difficult to remove surgically or for the treatment of disseminated metastases, systemic expression of this cytokine still remains as the most efficient method of administration. Nevertheless, finding alternative approaches for the use of IL-12 in the treatment of cancer and unraveling the basis of IL-12-side effects remain a challenge. In the present work we demonstrate that systemic expression of IL-12 through hydrodynamic injection of IL-12 cDNA is able to induce different types of liver lesions associated with a toxic pathology. However we report here that hepatic toxicity is diminished and survival of mice enhanced in the absence of tumor necrosis factor alpha (TNFα). This observation is in contrast to several murine models and clinical trials that postulate interferon gamma (IFNγ) as the main cytokine responsible for IL-12 toxicity. Moreover, our work demonstrates that when IL-12 cDNA is co-injected with IL-18 cDNA or when mice are pre-treated with a low dose of IL-12 cDNA prior to receiving a high dose of IL-12 cDNA, systemic levels of TNFα are almost completely abrogated, resulting in improved survival and less hepatic damage. Importantly, abrogation of TNFα signaling does not affect the strong anti-tumor activity of IL-12. Thus, neutralizing TNFα with antagonists already approved for human use offers a promising approach to abrogate IL-12 side effects during the use of this cytokine for the treatment of cancer.

## Introduction

The immunomodulating and anti-angiogenic functions of IL-12 have provided the rationale for exploiting this cytokine as an anticancer agent. More than a decade ago, clinical trials administering IL-12 for the treatment of tumors, including T cell lymphoma [1], non-Hodgkin lymphoma [2], melanoma [3], ovarian cancer [4], Kaposi's sarcoma [5], and renal carcinoma [6] were initiated. Systemic IL-12 was shown to be capable of suppressing tumor growth, metastasis, and angiogenesis *in vivo* [7]–[11], but its use has been associated with significant dose- and schedule-dependent toxicity, representing a major obstacle to its clinical application [1], [3], [4], [6], [7]. Grade 1-4 toxicity, has inhibited the use of IL-12 as a cancer therapy. However, alternative approaches for the use of IL-12 are under intensive investigation in animal models of cancer and in recent clinical trials [12]–[16].

Several clinical trials utilizing IL-12 have demonstrated that increased levels of the inflammatory cytokine IFNγ were associated with different grades of hepatotoxicity and other toxic side effects [2], [4], [6], [17]. Moreover, in several murine models, the IFNγ expression induced after IL-12 administration was found to be responsible for most of the observed adverse effects [18]–[21].

Utilizing different murine tumor models, we have previously reported that systemic levels of IL-12 alone or together with IL-18, induced after hydrodynamic injection of the respective cDNAs, completely abrogated subcutaneous tumor growth and pulmonary and hepatic metastases [10]. Moreover, co-expression of IL-12+IL-18 was able to improve survival by diminishing IL-12-side effects [10]. Our previous work also demonstrated that IFNγ was partially responsible for the IL-12-side effects. Moreover, our data demonstrated that increased survival of IL-12+IL-18-treated mice is associated with early IL-10 production and a reduction of IFNγ and TNFα serum levels [10]. In this context, the present work demonstrates that by using hydrodynamic injection to induce systemic co-expression of IL-12+IL-18 cDNAs or by pre-injecting a low dose of IL-12 cDNA prior to a large dose of IL-12 cDNA, we observed significantly increased survival and diminished hepatotoxicity. Interestingly, both methods demonstrate that this effect is highly associated with abrogation of TNFα expression, an inflammatory cytokine that also plays a pathological role in sepsis shock and *in vivo* LPS treatment [22]–[25]. Together, the data presented in this work contributes to our understanding of the basis for IL-12-systemic side effects. Furthermore, we propose that depending upon the system used to induce systemic IL-12 expression; different toxic mediators could be the protagonists of adverse side effects. Moreover, data presented in this manuscript can serve as the basis for the development of new approaches to decrease IL-12 toxicity when designing future therapies for cancer treatment involving this cytokine.

## Materials and Methods

### Mice and cell lines

C57BL/6 (B6) mice, 6-10 weeks of age, were used and maintained under specific pathogen-free conditions. Inducible nitric oxide synthase (iNOS) knock out (KO), TNFα KO and TNFα receptor one (TNFαR1) KO mice on a C57BL/6 genetic background were purchased from the Jackson Laboratories, Bar Harbor, ME, USA. Animal care was provided in accordance with the procedures outlined in the Guide for the Care and Use of Laboratory Animals (NIH-Publication No. 86–23, 1985). The experimental protocols were approved by the Institutional Animal Care and Use Committee of Centro de Investigaciones en Bioquímica Clínica e Inmunología (CIBICI), Consejo Nacional de Investigaciones Científicas y Técnicas (CONICET). Our animal facility has obtained NIH animal welfare assurance (assurance no. A5802-01, Office of Laboratory Animal Welfare, NIH, Bethesda, MD, USA). In order to avoid animal suffering, mice were rapidly sacrificed by cervical dislocation. During intrasplenic (i.s.) tumor cell injection, a shorter time of surgery was applied and the incisions were made as minimal as possible during the procedure. During recovery from surgery, mice were placed in warm blankets and their eyes were hydrated with a saline solution. Animals were placed back in cages after total recovery from the surgery.

B16-F10 melanoma cells were obtained from American Type Culture Collection. The cell line was free of Mycoplasma infection and tested by PCR every 6 months. B16-F10 melanoma cells were cultured in DMEM containing 10% FBS, 100 U/ml penicillin, 100 µg/ml streptomycin, and nonessential amino acids at 37°C, 5% CO_2_.

### Hydrodynamic cDNA injections

The hydrodynamic gene transfer procedure has been described previously [10], [26]. Briefly, animals were injected in the tail vein in less than 8 seconds with the respective cDNAs dissolved in 1.6 ml of sterile 0.9% sodium chloride solution and divided into 3 groups, control: 5–15 µg of ORF empty vector control cDNA, IL-12: 5 µg of IL-12 cDNA (pscIL-12, p40-p35 fusion gene) and IL-12+IL-18: 5 µg of IL-12 cDNA (pscIL-12, p40-p35 fusion gene) plus 10 µg of IL-18 cDNA (pDEF pro-IL-18). All the expression plasmids utilize the human elongation 1-α promoter to drive transcription.

The *in vivo* effects observed upon cytokine induction are purely due to the expression of the cytokine cDNAs and not by LPS contamination. WT and TLR4 KO mice develop similar levels and kinetics of TNFα expression after IL-12 cDNA treatment thus indicating the absence of LPS in the cytokine cDNA preparation used for injection (data not shown).

### Spleen samples

For cytokines analysis of *in vitro* stimulated cells, spleens were smashed, depleted of red cells by incubation in ACK lysing buffer (Biowhittaker, Walkersville, MD), washed and resuspended in supplemented media (RPMI 1640, 10% FBS, 2 mM L-glutamine, 5×10^−5^ M 2-mercaptoethanol, 1 mM sodium pyruvate and nonessential amino acids). Cell suspensions were counted and stimulated *in vitro* with supplemented media, anti-CD3 coated antibody (Ab) (BD-Pharmingen, La Jolla, CA) at 2 µg/ml or LPS (Sigma-Aldrich, St. Louis, MO) at 1 µg/ml for 72 h in an incubator set at 37°C and 5% CO_2_.

### Cytokine and ALT assays

Sera were assayed for cytokine production by ELISA according to the manufacturers' instructions. The kits utilized were: mIL-10, mIFNγ, mTNFα (BD-Pharmingen, La Jolla, CA). Alanine aminotransferase (ALT) sera levels were determined using a specific colorimetric kit (Wiener Laboratories) and by following the manufacturer's instructions.

### Hystopathological analysis of hepatic tissue

Livers obtained from mice in the different treatment groups were harvested on day 15 post cDNA treatment, fixed in 4% formaldehyde and then embedded in paraffin. 6 µm sections were prepared and hematoxilin/eosin (H/E) or Periodic acid-Schiff (PAS) stains were applied to the sections. Sections, without identification, were analyzed for tissue alterations under an optical microscope by a pathologist.

### Flow cytometry analysis

For multicolor staining, fluorocrome-conjugated Abs (BD-Pharmingen, La Jolla, CA) against lineage markers were used in various combinations. Briefly, Fc receptors on cells were blocked with an anti-CD16/32 Ab for 30 min at 4°C and washed. Cells were then stained for surface markers for 30 min at 4°C, washed twice and analyzed by flow cytometry with a BD FACS Canto™ II cytometer (BD Biosciences, San José, CA, USA). For flow cytometry cell sorting, splenocytes from IL-12+IL-18-cDNA treated mice were stained with fluorocrome-labeled CD4 (clone RM4-5), CD8 (clone 53-6.7), CD11b (clone M1/70) and CD11c (clone HL3) antibodies (BD-Pharmingen, La Jolla, CA) and sorted to a purity of 97–99% using a MoFlo sorter (Dako Cytomation).

### mRNA extraction and analysis

Total RNA was isolated using a single-step phenol/chloroform extraction procedure (TrIzol; Invitrogen Life Technologies). RT-PCR was performed with 100 ng of total RNA for each sample (Super Script III one step RT-PCR with platinum Taq, Invitrogen), consisting of a 15 minute reverse transcription reaction at 45°C, 40 cycles of denaturing at 94°C (15 s), annealing at 55°C (30 s) and extension at 68°C (60 s), with a final extension for 5 min at 68°C. Primers used were: iNOS, sense 5′-GCA TTT GGG AAT GGA GAC TG-3′, antisense 5′-GTT GCA TTG GAA GTG AAG CGT TTC-3′.

### Western immunoblotting

Splenocytes were lysed with 150 µl ice-cold lysis buffer and centrifuged at 14,000 rpm, 4°C for 5 min. Proteins extracts were electrophoresed on a 10% SDS-PAGE gel and transferred onto an Immun-Blot PVDF Membrane (BIO-RAD, Hercules, CA). Membranes were blocked with 5% milk, 0.1% Tween-20 in TBS overnight at 4°C and then were incubated with primary antibodies for 3 h at room temperature. After incubation with a horseradish-peroxidase conjugated secondary antibody (Cell Signaling Technology, Inc. Beverly, MA), the protein bands were detected with a Super Signal Chemiluminescent Substrate (Pierce) and BIOMAX-MR film (Eastman Kodak, Rochester, NY).

### Arginase Activity Assay

Triton X-100 (0.1%, 100 µl) was added to the splenocyte pellets that were then treated as indicated. After 30 min incubation, Tris-HCl and MnCl_2_ mixture (100 µl) were added to cells for 10 min at 56°C. The plates were incubated with L-arginine (100 µl) at 37°C for 30 min, and then an H_2_SO_4_/H_3_PO_4_/H_2_O mixture (800 µl) was added and heated with α-isopropylidene nitrobenzene acetone (50 µl) at 95°C for 30 min. The complex in each well was diluted 20 times with PBS to detect the optical density (OD) values at 540 nm with a UV spectrophotometer.

### Hepatic metastasis assay and subcutaneous tumor growth

C57BL/6 and TNFαRI KO mice were i.s. injected with B16-F10 melanoma cells (0.5×10^6^ cells/0.5 ml 0.9% sodium chloride solution) for hepatic metastasis analysis. Three days later, cDNAs were delivered by hydrodynamic injection. Eleven days later, livers were collected and the number of metastases was determined under a dissecting microscope.

To evaluate subcutaneous tumor growth, C57BL/6 and TNFαRI KO mice were shaved and injected s.c. in the left flank with 1×10^6^ B16-F10 melanoma cells in 0.2 ml of a sterile 0.9% sodium chloride solution. After 10–14 days, when solid tumors were visible (4–5 mm diameter), mice were hydrodynamically injected with the designated cDNAs. Tumor growth was monitored with a caliper. Day 0 corresponds to the day when cDNAs were administrated.

### Statistical analysis

For statistical significance, data was analyzed by means of a Student unpaired *t* test (comparison of 2 experimental groups) or ANOVA test (composition of more than 2 experimental groups) with p<0.05 considered as significant. Non-parametric test (Kruskal-Wallis test) was utilized when *n* was less than 10. Mouse survival curves were plotted as Kaplan – Meier plots using GraphPad Prism Version 4 (GraphPad Software, San Diego, CA) and a p<0.05 was considered as significant.

## Results

### Changes in leucocytes numbers after systemic expression of IL-12 or IL-12+IL18

**Figure 1:**
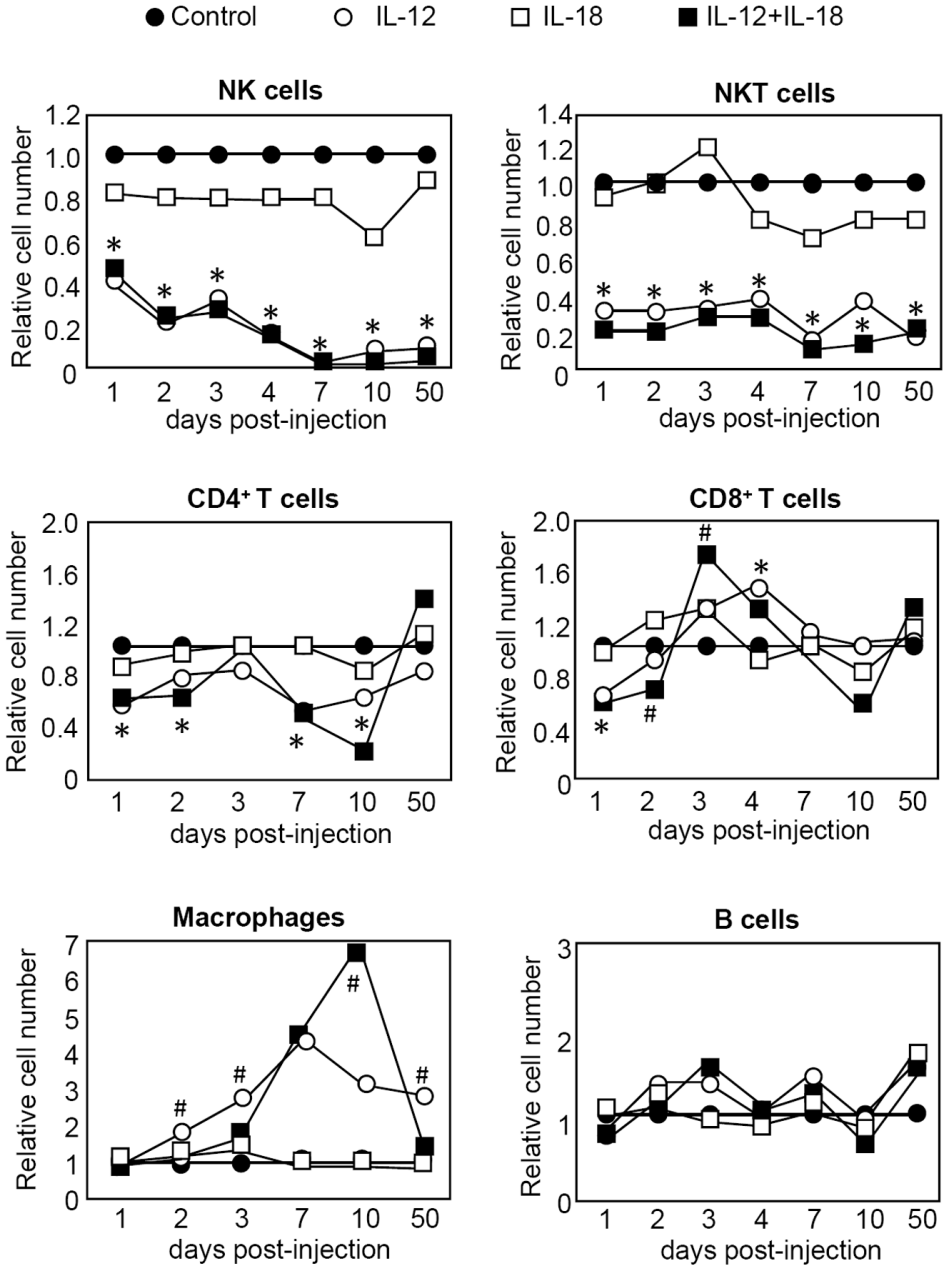
Relative absolute cell numbers of splenic cell subpopulations in mice hydrodynamically injected with cytokine cDNAs. C57BL/6 mice were hydrodynamically injected with control, IL-12 and/or IL-18 cDNAs. Up to 50 days post-injection, animals were sacrificed and single cells suspensions of splenic cells were obtained. The percentage of the different subsets was obtained by surface lineage staining with combination of the following Abs: NK1.1, CD3, CD11b, CD19, CD4 and CD8 and analyzed by flow cytometry. Relative cell numbers of the different cell populations were calculated by dividing the absolute cell numbers obtained from the cytokine-treated mice by the absolute cell numbers obtained from control mice. Data is expressed as the mean of a pool of 4 different experiments (total n = 12 mice per group). *12+18 and 12 vs control p<0.05, ^#^12 vs 12+18 p<0.05.

We have previously demonstrated that depletion of T cells and NK/NKT cells increases survival of IL-12-treated mice [10]. However, a residual toxicity persists after T/NK/NKT cell depletion, suggesting that other cell types could also be part of the IL-12-adverse effects [10]. As a first step to investigating this issue we analyzed the relative changes in the numbers of leucocytes present in the spleen (and liver, not shown) compared to control mice. As seen in Figure 1, the numbers of NK and NKT subsets are highly reduced up to 50 days post-treatment following administration of either IL-12 or IL-12+IL-18 cDNAs. This data seemed quite surprising since depletion of NK/NKT cells correlates with improvement in survival of mice treated with IL-12 cDNA. However, when we evaluated the phenotype of NK cells obtained from IL-12-treated mice, we observed that they express high levels of IFNγ and the early activation marker CD69 (data not shown). This data suggests that even though they are found in very low numbers, NK/NKT cells demonstrate an activated phenotype and thus may be partially responsible for the toxicity observed after systemic expression of IL-12.

We also observed an alteration in the number of T lymphocytes following cytokine cDNA administration. While CD4^+^ T cells show a lower cellularity during most time periods analyzed, CD8^+^ T cells undergo an expansion in numbers during the first week after treatment but then subsequently return to normal levels (fig.1).

Next we evaluated the macrophage (Mφ) population in the treated mice. IL-12 alone induces an early increase in Mφ numbers while in contrast, the number of these cells in IL-12+IL-18-treated mice is not expanded (days 2 and 3 post-treatment). Moreover, even though Mφ numbers reach a higher peak in IL-12+IL-18 treated mice by day 10, they return to normal levels by day 50 post-treatment while the number of Mφ in the IL-12 treated group is still elevated. The fact that a pathogenic role for these cells in another murine model of IL-12+IL-18 systemic expression has been previously reported [19], we hypothesize that mediators produced by Mφ could be responsible, at least in part, for the toxic side effects observed after systemic IL-12 treatment.

### The balance between nitric oxide (NO) expression and arginase activity suggests a more inflammatory profile in macrophages from IL-12-treated compared to IL-12+IL-18-treated mice

**Figure 2:**
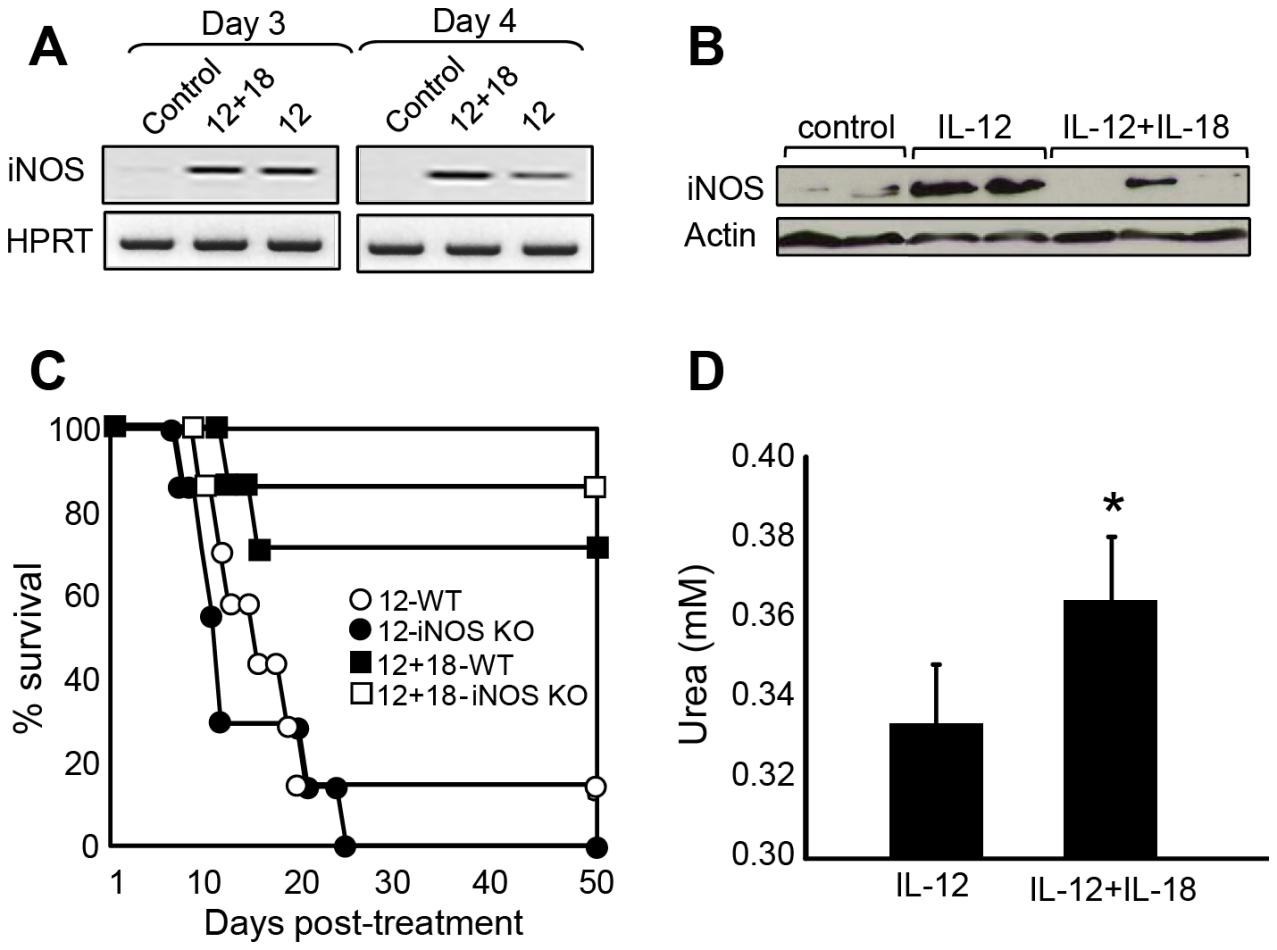
inflammatory profile of Mφ following IL-12 cDNA treatment. (**A**) RT-PCR analysis of iNOS gene expression in splenocytes obtained from mice injected with the designated cDNAs, 12+18 and 12 vs control p<0.05. (**B**) Western blot analysis of splenocyte protein extracts obtained from mice injected with the designated cDNAs, 12 vs 12+18, p<0.05. (**C**) Survival monitored in B6 (WT) or iNOS KO mice in the days post-IL-12 or IL-12+IL-18-cDNA injection. (**D**) Arginase activity measured determination of urea levels in 1×10^6^ splenocytes. The data in A, B and D are expressed as the mean of 2 different experiments with 4–5 mice per group. Survival curve was built as a pool of 2 different experiments with 5 mice per group. *12+18 vs 12 p<0.05.

Next, we investigated the role of NO as a basis for IL-12-side effects as NO is known to be induced by Mφ in the presence of IL-12+IL-18 [27]. The generation of NO by neutrophils and monocytes is increased in septic patients and their persistence is associated with a poor clinical outcome [28]. Moreover, excessive NO formation plays an important role in the pathogenesis of shock and multiple organ failure during sepsis and in acute lung injury [29]. In this context, we first analyzed if NO/iNOS is expressed in our experimental model. As can be seen in Figure 2A, iNOS RNA expression is significantly up-regulated in both IL-12 and IL-12+18-treated mice early after cDNA administration and persists for at least 15 days after treatment (data not shown). Interestingly, protein analysis revealed that after 15 days post-treatment, iNOS expression is still up-regulated in IL-12-treated mice but lower or no expression is observed in IL-12+IL-18-treated mice (Fig. 2B). Moreover, *in vitro* NO levels evaluated on day 15 post treatment from splenocytes stimulated with LPS showed a lower expression in cells obtained from IL-12+IL-18-treated mice (data not shown). These data led us to determine if the lower expression of iNOS/NO in IL-12+IL-18-treated mice could explain the improved survival outcome observed in this group, as compared to IL-12-treated mice [10]. Surprisingly, when we monitored survival in WT and iNOS KO mice after IL-12 or IL-12+IL-18 cDNA administration, we observed that the lack of iNOS expression does not improve resistance to the treatments (Fig. 2C). These results led us to hypothesis that NO might not be directly involved as a toxic mediator of these treatments but the relation between Mφ NO/arginase expression could be providing information about the inflammatory environment resulting from the different treatments. Accordingly, figure 2D shows that splenocytes from IL-12-treated mice have significantly lower arginase activity when compared to splenocytes from IL-12+IL-18-treated mice. Together with the iNOS data, we conclude that the NO/arginase ratio is higher in mice treated with IL-12 alone than in mice treated with IL-12+IL-18. This result suggests that the treatment with IL-12+IL-18 induced a macrophage phenotype less inflammatory when compared to IL-12 treated mice.

### Expression of IFNγ, TNFα and IL-10 after IL-12+IL-18 treatment

**Figure 3:**
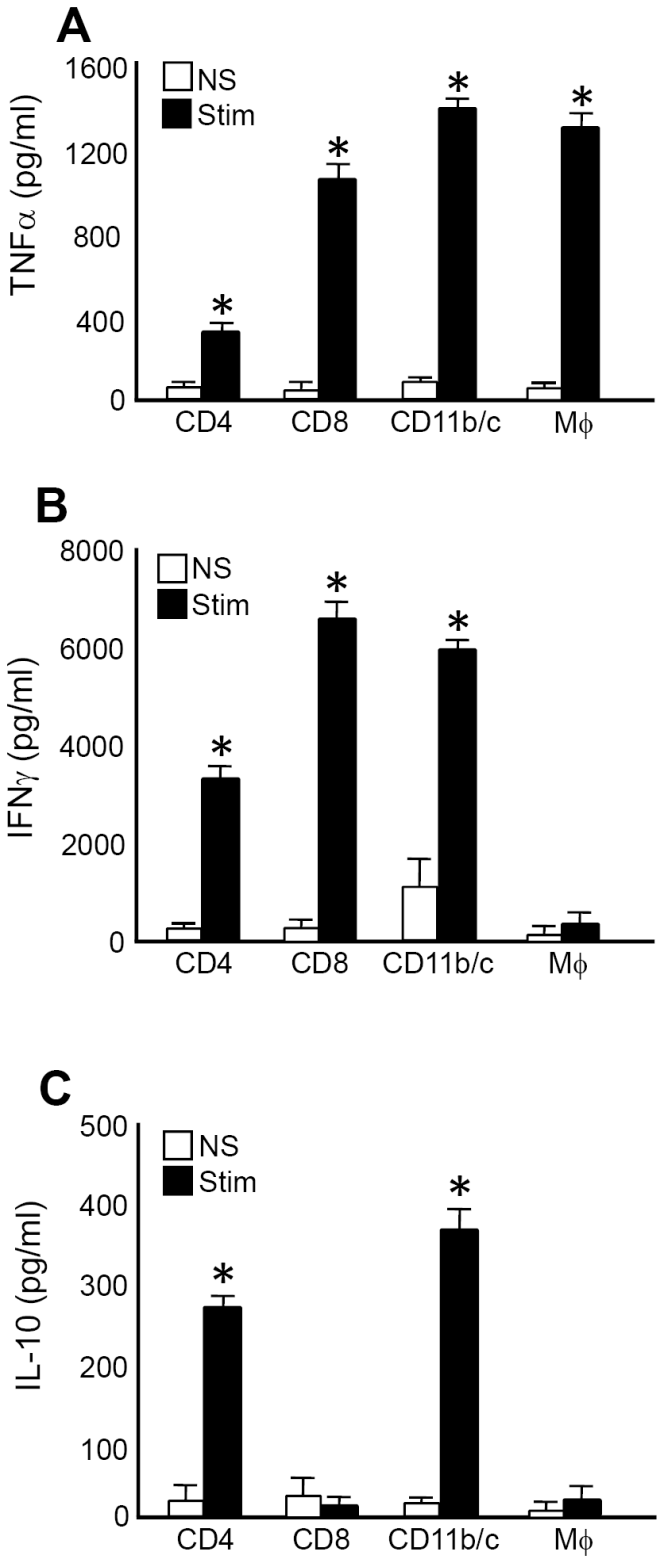
Splenocytes produce Th1 and Th2 cytokines after IL-12+IL-18 cDNA treatment. Splenocytes from IL-12+IL-18 mice were obtained and stained with anti-CD4, anti-CD8 and anti-CD11b/c fluorocrome labeled Abs and then sorted to a purity of 97–99% using a MoFlo sorter (Dako Cytomation). Sorted cells were then cultured in the presence of anti-CD3 coated Ab (2 µg/ml) and LPS (1 µg/ml) for 72 h. Supernatants were then harvested and (**A**) TNFα, (**B**) IFNγ and (**C**) IL-10 expression were evaluated by ELISA. Data are expressed as the mean of 2 different experiments with 4-5 mice per group. *Stimulated vs non-stimulated, p<0.001.

We have reported that in IL-12+IL-18-treated mice, early expression of IL-10 controls the levels of the inflammatory cytokines IFNγ and TNFα [10]. However, even though we performed these experiments by depleting different leucocytes populations and evaluating survival in each case, we could not identify the sources of the cytokines [10]. To investigate this issue, we evaluated the production of IL-10, IFNγ and TNFα from sorted splenocytes obtained from IL-12+IL-18-treated mice on day 3 post treatment, a timepoint at which we had previously observed production of these cytokines (Figure 3). We observed that TNFα is highly produced by CD8^+^ T cells and CD11b^+^ plus CD11c^+^ cells (Mφ, dendritic cells (DCs) and natural killer (NK) cells sorted together). Since NK cells and DCs represent a very small population of splenocytes in these mice (Fig. 1 and data not shown), we sorted all these cells together to increase the number of recovered cells. To determine the contribution of Mφ alone, we evaluated the cytokine expression in cultures of enriched adherent splenocytes (>85% Mφ) and observed that these cells also expressed high levels of TNFα. This observation suggested that the expression of TNFα may come mainly from Mφ rather than the other 2 cell types present in the CD11b/c^+^ sorted group (Fig. 3A). In contrast, IFNγ is expressed by CD4^+^ and CD8^+^ T cells but not from Mφ, demonstrating that CD11b/c expression is probably due to the presence of NK cells in this group (Fig. 3B). Interestingly, IL-10 is produced by CD4^+^ T cells and also by DCs or NK cells but not by Mφ (Fig. 3C). This data suggests that depletion of T, NK and NKT cells, could eliminate the sources of IFNγ (as we previously reported [10]), but Mφ still remain as a very important source of TNFα. This finding is probably why depletion of the lymphocytes only partially improved survival in IL-12-treated mice [10]. Moreover, the fact that the Mφ profile seems to be more inflammatory based on the NO/arginase data, could explain why IL-12-treated mice exhibit much lower survival than IL-12+IL-18-treated mice.

### TNFα is an important cytokine that mediates IL-12 systemic toxicity

**Figure 4:**
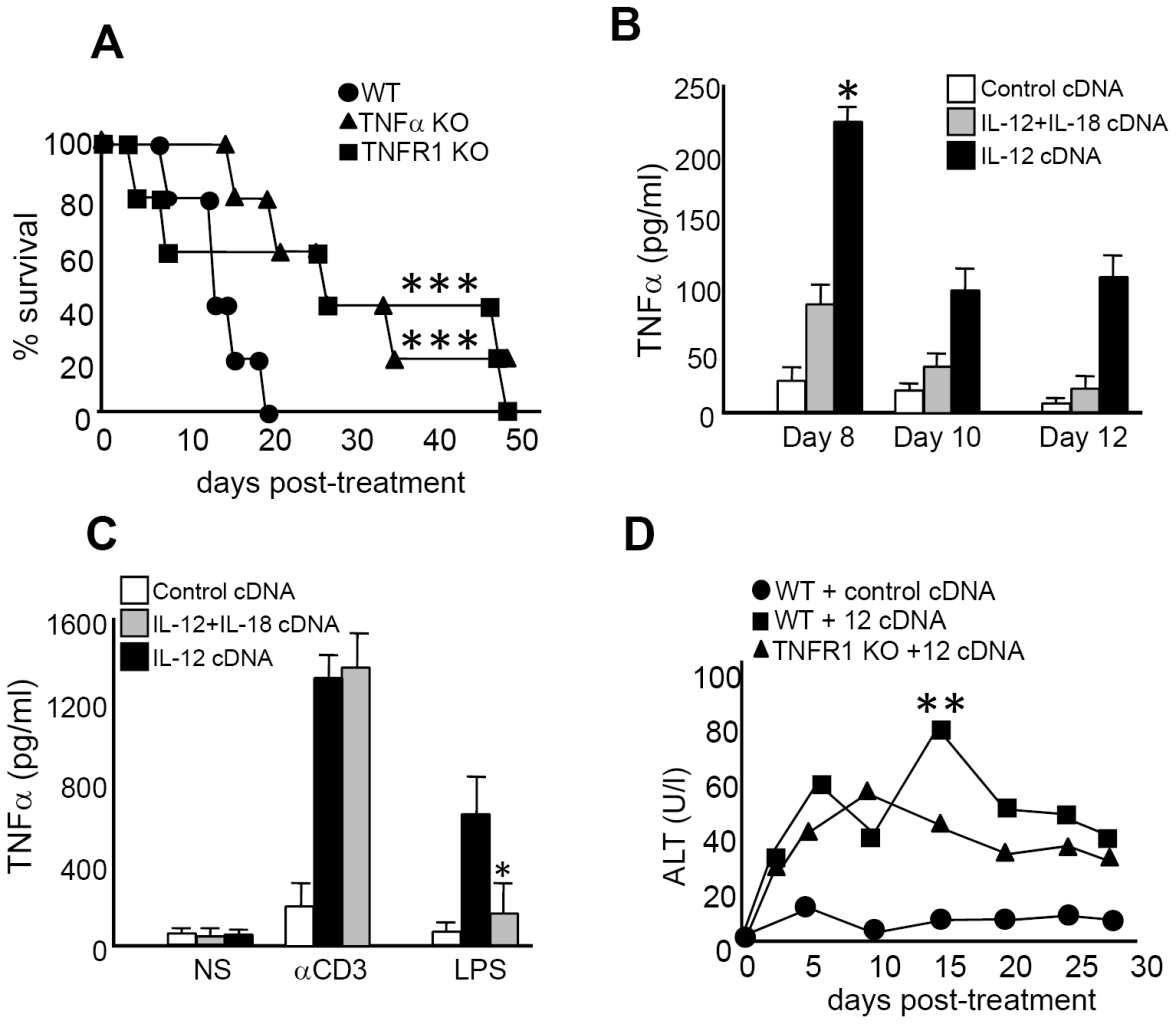
TNFα plays a critical role in mediating IL-12 systemic adverse effects. (**A**) Survival was monitored in WT, TNFα KO and TNFRI KO mice in the days post-IL-12 cDNA treatment. *WT vs TNFα KO and TNFRI KO, p<0.01. (**B**) Mice from control, IL-12 or IL-12+IL-18-cDNA treated groups were bled and TNFα sera levels were determined in the days post-treatment. (**C**) Splenocytes from control, IL-12 or IL-12+IL-18 cDNA treated mice were obtained and cultured in the presence of media (NS), anti-CD3 coated Ab or LPS for 72 h. Supernatants were then harvested and TNFα expression was evaluated by ELISA. *IL-12 vs IL-12+IL-18, p<0.05. (**D**) Sera from B6 mice treated with control or IL-12 cDNA and from TNFRI KO mice treated with IL-12 cDNA were obtained in the days post-treatment. ALT levels were determined using a specific colorimetric kit. The data in A, B and C are expressed as the mean of 2 different experiments with 4 mice per group. The data in D are expressed as the mean of the pool of 3 different experiments (WT n = 12, TNFR1 KO n = 9). *WT IL-12 cDNA vs TNFRI KO IL-12 cDNA, p<0.05.

To investigate the role of TNFα as a toxic mediator after IL-12 systemic expression, we analyzed survival of TNFα KO or TNFαR1 KO mice after IL-12 treatment. As seen in Figure 4A, abrogation of TNFα or its type 1 receptor genes significantly increased survival of IL-12-treated mice compared to WT mice. Interestingly, when we analyzed the kinetics of TNFα serum levels, we observed that protein expression peaks around day 4 both in IL-12+IL-18- and in IL-12-treated mice; however, the levels are much lower in IL-12+IL-18-treated mice (Fig. 6C). Moreover, by day 8 and up to day 12 post-treatment, TNFα sera levels begin to decline in both groups; however, it remains persistent and always higher in the IL-12 treatment group (Fig. 4B).

Next, we compared TNFα *in vitro* expression by splenocytes obtained from the mice treated *in vivo* with IL-12 alone or with IL-12+IL-18. While TNFα produced after *in vitro* stimulation with an anti-CD3 Ab (stimulation of T cells) is quite similar between cells obtained from both groups, it is significantly lower in IL-12+IL-18-treated mice when stimulated *in vitro* with LPS (i.e. Mφ and DCs stimulation) (Fig. 4C). This data suggests that the lower levels of systemic TNFα observed in IL-12+IL-18-treated mice is probably due to a reduced contribution of Mφ and DCs in the expression of this cytokine in this treatment group.

### Systemic IL-12 or IL-12+IL-18 treatment provokes pathological effects in immune tissues and the liver

Systemic expression of IL-12 has been reported to induce several types of pathological changes in mouse models and in cancer patients [2], [4], [6], [17]. To evaluate the general toxicity of IL-12 treatment, we sacrificed control and sick IL-12 and IL-12+IL-18 treated mice and performed a complete analysis of organ pathology. As can be seen in Table S1, the major alterations observed are atrophy of the thymus and lymphoid depletion, and these changes appeared similar in IL-12 and IL-12+IL-18 treated mice.

However, the analysis also revealed a more intense histiocytosis in lymph nodes (LNs) obtained from IL-12-treated mice compared to IL-12+IL-18-treated mice. Histiocytosis is associated with the presence of macrophages in the tissue, so the exacerbated pathology in IL-12 treated mice is associated with a major expansion/presence of macrophage in LNs.

### Alterations in hepatic tissue after systemic expression of IL-12 or IL-12+IL-18

Even though we did not observe differences in histiocytosis when comparing the livers of IL-12 and IL-12+IL-18 mice, previous results confirmed that sera levels of the hepatic enzymes ALT and aspartate aminotransferase (AST) were significantly higher in IL-12-treated mice [10]. Next we decided to evaluate the pathology of this organ in lieu of the finding that one of the most undesirable effects of systemic IL-12 therapy is the hepatotoxicity observed after the administration of this cytokine [2], [4], [6], [17]. Interestingly, as shown in Figure 4D, IL-12 treatment of TNFα type 1 receptor KO mice abrogates the ALT peak observed by day 15 in WT mice.

**Figure 5:**
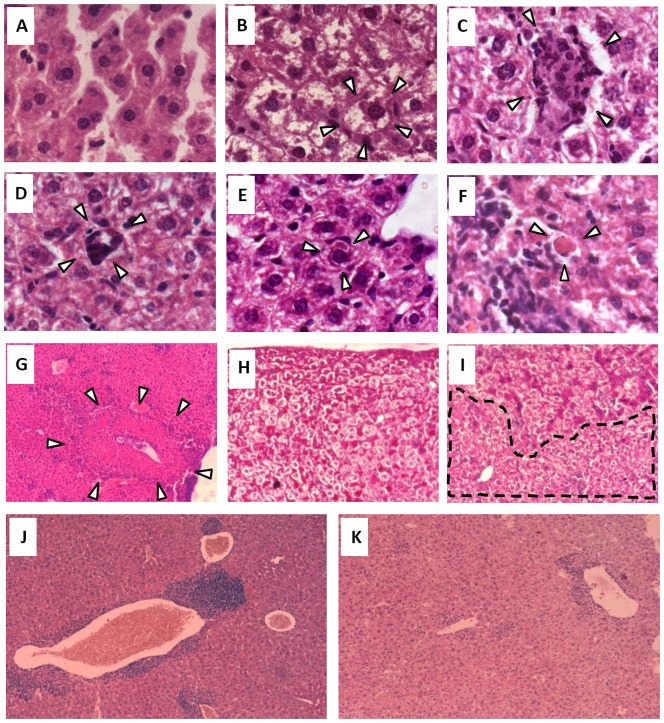
Histopathological alterations in hepatic tissue after systemic expression of IL-12 or IL-12+IL-18. Livers obtained from mice in the different groups were obtained on day 14-16 post-cDNA treatment. Sections of 6 µm were obtained and (**A–G**) hematoxilin/eosin (H/E) or (**H–I**) Periodic acid-Schiff (PAS) stains were applied to the sections. (**A**) Hepatocytes and sinusoids from a control mouse, 100×. (**B**) Ballooning hepatocytes and absence of hepatic sinusoids in an IL-12-cDNA treated mouse, 100×. (**C**) Focus of ectopic hematopoiesis in an IL-12-cDNA treated mouse, 100×. (**D**) Megakaryocyte in an IL-12-cDNA treated mouse, 100×. (**E**) Mallory and (**F**) Councilman bodies in an IL-12-cDNA treated mouse, 100×. (**G**) Focus of necrosis; 10×. Glycogen deposits obtained in a mouse from (**H**) the control group or (**I**) the IL-12-cDNA group, 20×. Liver sections from (**J**) WT or (**K**) TNFR1 KO mice 15 days after IL-12 cDNA treatment. Pictures are representative of 2 different experiments with 4–5 mice per group.

The histopathological features present in the livers of mice treated with IL-12 or IL-12+IL-18 cDNAs revealed major changes in the tissue, including the appearance of: cells with a round shape (cell ballooning) (Fig. 5B) compared to the classical hexagonal shape of hepatocytes from control mice (Fig. 5A). Moreover, the livers from treated mice show the loss of the hepatic sinusoids that can be clearly observed in control mice (Figs. 5A vs 5B). Additionally, we observed foci of ectopic hematopoiesis (Fig. 5C) with the presence of megakaryocytes (Fig. 5D) throughout the liver in IL-12- or IL-12+IL-18-treated mice but not in control mice. Both Mallory bodies (Fig. 5E) and Councilman bodies (Fig. 5F) and foci of necrotic tissue (Fig. 5G) can only be seen in mice treated with the cytokine cDNAs. Finally, we observed that glycogen deposits are highly reduced in mice treated with IL-12 or IL-12+IL-18 (Fig. 5I) compared to control mice (Fig. 5H). Even though all these lesions are present throughout the liver tissue in mice treated with either IL-12 cDNA alone or IL-12+IL-18 cDNAs (peaking between days 14–16), co-administration of IL-12 and IL-18 is able to reduce the incidence of these IL-12-associated hepatic abnormalities (Table S2). These observations correlate with our previous data that demonstrated lower ALT and AST sera levels in 12+IL-18-treated mice [10]. Moreover, TNFαR1 KO mice (Fig. 5K) exhibited a lower incidence of these pathological features after IL-12 cDNA treatment than what was observed in treated WT mice (Fig. 5J), especially in the number and size of infiltrate foci (Table S2).

**Figure 6:**
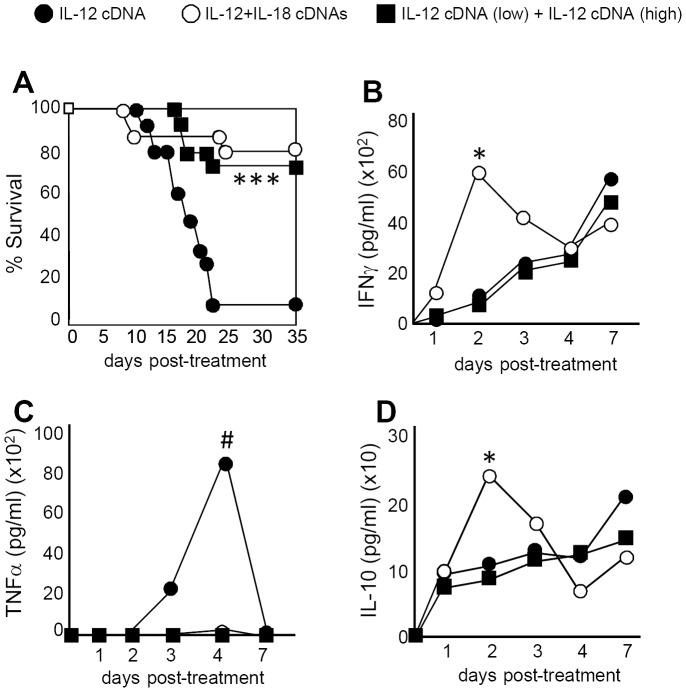
IL-12+IL-18 co-expression and low amounts of IL-12 cDNA reduce IL-12-side effects by decreasing TNFα expression. B6 mice were treated with IL-12 cDNA alone (black circle), IL-12+IL-18 cDNA (open circle) or low IL-12 cDNA (0,5 µg) 2 days prior to administration of a high amount of IL-12 cDNA (5 µg) (black square). (**A**) Survival was monitored and (**B**) IFNγ, (**C**) TNFα and (**C**) IL-10 sera expression were evaluated by ELISA in the days post-treatment. The data are expressed as the mean of 2 different experiments with 5 mice per group. IL-12+IL-18 or IL-12 low cDNA + IL-12 high cDNA vs IL-12, ***p<0.01 or ^#^p<0.05. IL-12+IL-18 vs the rest of the groups, *p<0.05.

### Correlation of TNFα adverse effects with clinical data in cancer patients treated with IL-12

Clinical trials results with IL-12 protein administration and in murine models demonstrated that a low dose of IL-12 given prior to a larger dose of IL-12 (dose escalating protocols) prevents major toxicity by the high dose IL-12 [17], [30]–[32]. Therefore the question arises as to whether pre-dosing with a low dose of IL-12 in our model might have the same effect as co-administration of IL-18 cDNA, and if so, might the treatment regime also work via early IL-10 induction and attenuation of TNFα and IFNγ levels.

To test this hypothesis, we pre-injected mice with a low dose of IL-12 cDNA followed 2 days later by a high dose of IL-12 cDNA. We evaluated serum expression of IL-10, TNFα and IFNγ in this group and compared the data with mice injected with IL-12 cDNA alone or IL-12+IL-18 cDNA. As we previously reported [10], a significant increase in survival can be observed in IL-12+IL-18 cDNA-treated mice compared to mice treated with IL-12 cDNA alone (Fig. 6A). Interestingly, when mice were pre-injected with a low dose of IL-12 cDNA, a significant increase in survival is detected similar to what is observed in IL-12+IL-18-cDNA treated mice. These data encouraged us to investigate if pre-dosing with IL-12 is able to trigger a similar protective mechanism as observed when co-injecting with IL-12+IL-18 cDNA. We analyzed cytokine sera levels in all groups of mice up-to 7 days post-treatment and interestingly, IFNγ and IL-10 serum levels expression is up-regulated early after treatment only in IL-12+IL-18 cDNA treated mice but not in the rest of the groups (Fig. 6B). Furthermore, mice that demonstrate a better survival (IL-12+IL-18 and pre-low dose IL-12 cDNA groups) are also in the groups that show very low levels of TNFα in the sera. In contrast, elevated sera TNFα correlates with a poor outcome in IL-12 cDNA-treated mice (Fig. 6A and 6C). It is important to clarify that IL-12 serum levels induced after the different treatments are similar (data not shown), thus the differences in TNFα levels cannot be attributed to differences in IL-12 expression following hydrodynamic injection.

### Blocking TNFα signaling ameliorates IL-12-side effects without affecting the IL-12 antitumor capacity

**Figure 7:**
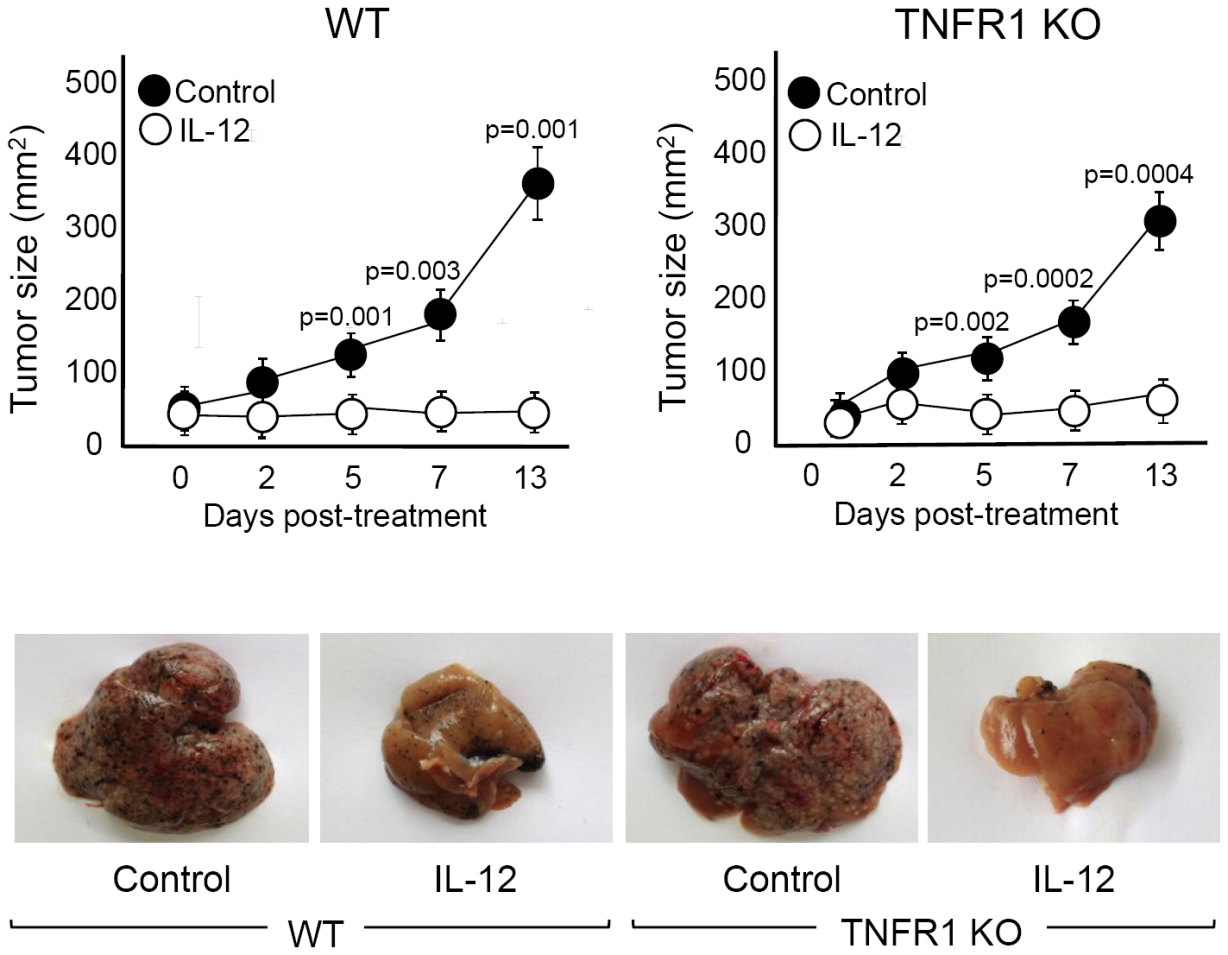
Abrogation of TNFα signaling does not affect IL-12 antitumor activity. For subcutaneous tumor growth, 1×10^6^ B16 cells were injected s.c. in C57BL/6 (WT) and TNFR1 KO mice (8–10 weeks old). Between days 10–14, when tumors had reached a size of approximately 5 mm, mice were hydrodynamically injected with IL-12 cDNA. In the days post cDNA injection, tumor growth was measured with a caliper (upper panel). For liver metastases analysis, intrasplenic injection was performed on day 0 with 0.5×10^6^ B16 melanoma cells and 3 days later with IL-12 cDNA via hydrodynamic shear. Liver examination was performed on day 14 post treatment (lower panel). IL-12 vs control p values are plotted in the figure. The data in the upper panel are expressed as the mean of 2 different experiments with 4 mice per group. Pictures of the lower panel are representative of 2 different experiments with 4–5 mice per group.

The data we have presented demonstrates a pathological role for TNFα after systemic expression of IL-12. Moreover, we have proven that incorporating alternatives to IL-12 systemic administration (IL-12+IL-18 and pre-low dose IL-12 cDNA) highly ameliorates IL-12-side effects by reducing TNFα sera levels. In order to prove that the blockage of TNFα signaling could be considered for cancer patients treated with IL-12, we must demonstrate that abrogation of TNFα does not affect the powerful antitumor capacity of IL-12. In order to test this hypothesis, we evaluated the antitumor effect of IL-12 in WT and TNFR1 KO mice utilizing the B16 melanoma subcutaneous tumor and liver metastases murine models. Similar to our previous report [10], we found that administration of IL-12 cDNA almost completely abrogates the growth of B16 s.c. tumors either in WT or TNFR1 KO mice (upper panel of Figure 7). Moreover, after IL-12 treatment, both strains of mice show lower hepatomegaly and a reduction in the size and number of B16 hepatic metastases (Figure 7, lower panel).

As a whole, the data presented in this work demonstrate an important role of TNFα in the toxicity mediated by systemic expression of IL-12. We can conclude that different treatment protocols that lead to a control of TNFα levels (e.g. treatment with anti-TNF antibodies or with soluble TNF receptor) warrant consideration when designing new cancer therapies utilizing IL-12, without risking its potent antitumor activity.

## Discussion

Eradication of residual malignancies and metastatic tumors via a systemic approach is the key for successfully treating cancer and increasing cancer patient survival. Several clinical trials in patients with different type of cancers have demonstrated that systemic administration of IL-12 protein in a large acute dose is effective in only a percentage of the patients but toxic in most of patients [1]–[6]. However, the strong anti-angiogenic properties of IL-12, together with its capacity to mount a powerful T cell immune response against the tumor are still being exploited in recent clinical trials [12], [13]. Moreover, in order to diminish IL-12 toxicity, several laboratories have attempted to deliver IL-12 to the site of the tumor, with promising results [1], [33], [34]. However, in the case of metastases or tumors difficult to access, systemic IL-12 administration still represents the best treatment option. Thus, the development of alternatives to IL-12 systemic use and identifying the molecules responsible for its side effects will contribute to expanding the use of IL-12 in the treatment of cancer.

In this context, our previous data showed that co-expression of IL-12 plus IL-18 cDNA achieved strong anti-tumor effects, similar to IL-12 cDNA alone, along with a significant increase in survival [10]. This observation was quite surprising as it has been previously reported that a synergistic, albeit toxic, effect was observed when both cytokines were co-administrated as recombinant proteins [19], [35]. In order to understand the mechanism of toxicity in this model and how co-expression of both cytokines induced benefits in tolerating the treatment, we previously analyzed IFNγ expression as this cytokine was reported to play an important role in mediating IL-12-side effects [18], [19], [35]. Consistent with this hypothesis, most experimental models and clinical trials point to IFNγ as the cytokine involved in IL-12-side effects [7], [18], [19], [35]. However, in the present model of cDNA administration that results in systemic expression of IL-12+IL-18, we have previously reported an ambiguous role for IFNγ, as its reduction improved survival but its complete abrogation induced 100% death of mice treated with IL-12 or IL-12+IL-18 (probably due to pulmonary edema) [18], [35]. Taking this data into account, we focused our attention on identifying other possible toxic mediators responsible for IL-12 side effects. We present evidence here that TNFα acts as a critical cytokine that mediates IL-12-adverse effects. Consistent with our findings, TNFα has been described as the major mediator in the pathogenesis of other inflammatory conditions, including septic shock [22]–[25].

Our previous data demonstrated that simultaneous depletion of T and NK cells significantly ameliorates survival in mice treated with IL-12 cDNA [10]. With this data in mind, we speculate that this effect is due to abrogation of the different sources of TNFα. Interestingly, data from the present work demonstrates that TNFα production is observed in T cells, NK cells and also in Mφ. This residual Mφ TNFα expression is probably why we observed that a percentage of mice depleted of NK-T cells still succumb following IL-12 cDNA treatment [10]. Moreover, we demonstrate here that when comparing NO vs arginase levels together with TNFα production, Mφ from IL-12+IL-18-treated mice seems to exhibit a lower inflammatory profile when compared to IL-12-treated mice.

Hepatotoxicity is one of the most undesirable side effects observed after systemic IL-12 treatment and yet the consequences of TNFα systemic expression on liver pathology has not been carefully studied [2], [4], [6], [17]. We report here several hystopathological alterations develop in mice treated with IL-12 or IL-12+IL-18 cDNA and these alterations have been previously associated with liver toxicity. These effects include Mallory bodies and hepatocytes ballooning as seen in alcoholic and non alcoholic hepatitis [36], [37] and necrosis and Councilman bodies observed in Dengue fever and hepatitis C virus infection [38], [39]. Moreover, further evidence of liver damage comes from the fact that we detected highly increased ALT and AST serum levels in the cytokine cDNA treated mice [10]. Interestingly, both the liver enzymes levels and the changes in the liver tissue are reduced in mice treated with IL-12+IL-18 cDNA compared to mice treated with IL-12 cDNA alone. Furthermore, we demonstrate here that abrogation of TNFα or its type 1 receptor reduces ALT levels and hepatic alterations and also improves survival of IL-12 cDNA treated mice. Additionally and consistent with our model, TNFα is almost absent from the serum in IL-12+IL-18 cDNA treated mice.

The data presented in this work points to TNFα more than IFNγ as a major pathological mediator resulting from systemic IL-12 expression. Somewhat surprisingly, these results are in contrast to several reports that conclude that IFNγ expression after IL-12 or IL-12+IL-18 administration is responsible for IL-12 toxicity [18], [19], [35]. Based on data from murine models and clinical trials [17], [30]–[32], the reduced toxicity observed after pre-treating with a low dose of IL-12 before administration of a high dose of IL-12 or treatment with both IL-12 and IL-18 cDNA, may act through the control of systemic TNFα levels.

Overall, the data presented in this manuscript contribute new insights into the clinical use of IL-12 by revealing strong evidence that TNFα may act as the main molecule responsible for IL-12-side effects. Based on the fact that abrogation of TNFα does not affect the potent IL-12 antitumor capacity, this work supports the development of alternatives approaches and therapies capable of reducing toxicity, including the addition of approved TNFα antagonists (infliximab, adalimumab, etc), when using IL-12 in cancer patients.

## Supporting Information

Table S1 **Pathology in mice treated with systemic IL-12 or IL-12+IL-18**. A complete body examination was performed by a pathologist in mice treated with control, IL-12 or IL-12+IL18 cDNAs. Table S1 shows the description only in the organs that present any pathological alterations.
(PPTX)

Table S2 **Hepatic pathology in mice treated with systemic IL-12 or IL-12+IL-18.** Quantitative analysis of the indicated pathological conditions in B6 or TNFαR1 KO mice treated with control, IL-12 or IL-12+IL-18 cDNAs. Infiltration  =  mononuclear cells and hematopoeisis. Cellular lesions  =  Cell balloning and Mallory and Councilman bodies. -  =  no pathology; +  = mild; ++  =  moderate; +++  =  marked
(PPTX)
