# Supplementary material for: Abrogation of TNFα Production during Cancer Immunotherapy Is Crucial for Suppressing Side Effects Due to the Systemic Expression of IL-12
Source: PLoS One. 2014 Feb 28;9(2):e90116. doi: 10.1371/journal.pone.0090116 (PMC3938584; doi:10.1371/journal.pone.0090116)
Supplement: Table S1 — Pathology in mice treated with systemic IL-12 or IL-12+IL-18. A complete body examination was performed by a pathologist in mice treated with control, IL-12 or IL-12+IL18 cDNAs. Table S1 shows the description only in the organs that present any pathological alterations. (PPTX) [file pone.0090116.s001.pptx]

## Slide 1
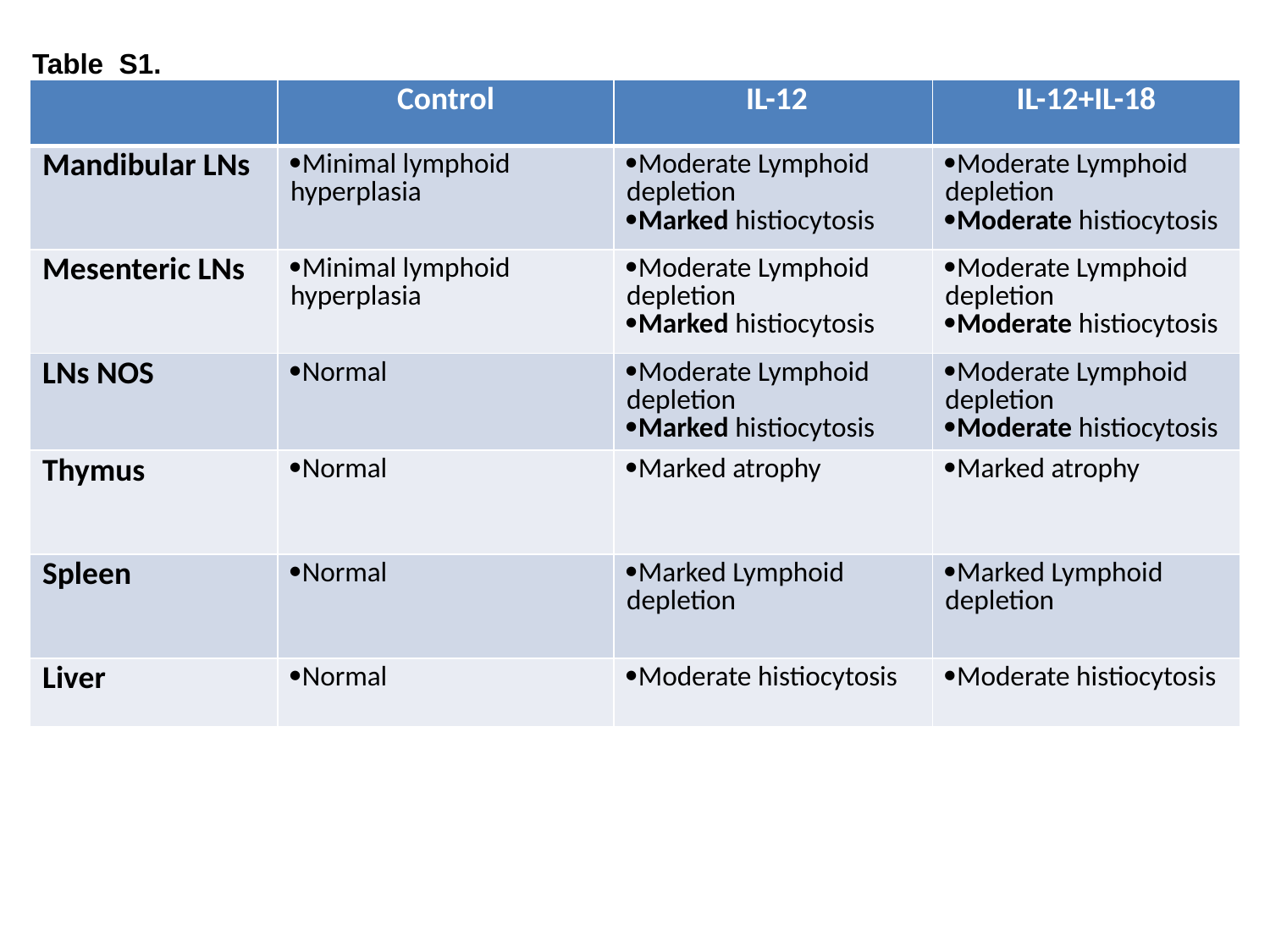

Table S1.
| | Control | IL-12 | IL-12+IL-18 |
| --- | --- | --- | --- |
| Mandibular LNs | Minimal lymphoid hyperplasia | Moderate Lymphoid depletion Marked histiocytosis | Moderate Lymphoid depletion Moderate histiocytosis |
| Mesenteric LNs | Minimal lymphoid hyperplasia | Moderate Lymphoid depletion Marked histiocytosis | Moderate Lymphoid depletion Moderate histiocytosis |
| LNs NOS | Normal | Moderate Lymphoid depletion Marked histiocytosis | Moderate Lymphoid depletion Moderate histiocytosis |
| Thymus | Normal | Marked atrophy | Marked atrophy |
| Spleen | Normal | Marked Lymphoid depletion | Marked Lymphoid depletion |
| Liver | Normal | Moderate histiocytosis | Moderate histiocytosis |
