# Supplementary figures and images for: Abrogation of TNFα Production during Cancer Immunotherapy Is Crucial for Suppressing Side Effects Due to the Systemic Expression of IL-12
Source: PLoS One. 2014 Feb 28;9(2):e90116. doi: 10.1371/journal.pone.0090116 (PMC3938584; doi:10.1371/journal.pone.0090116)

## Slide 1
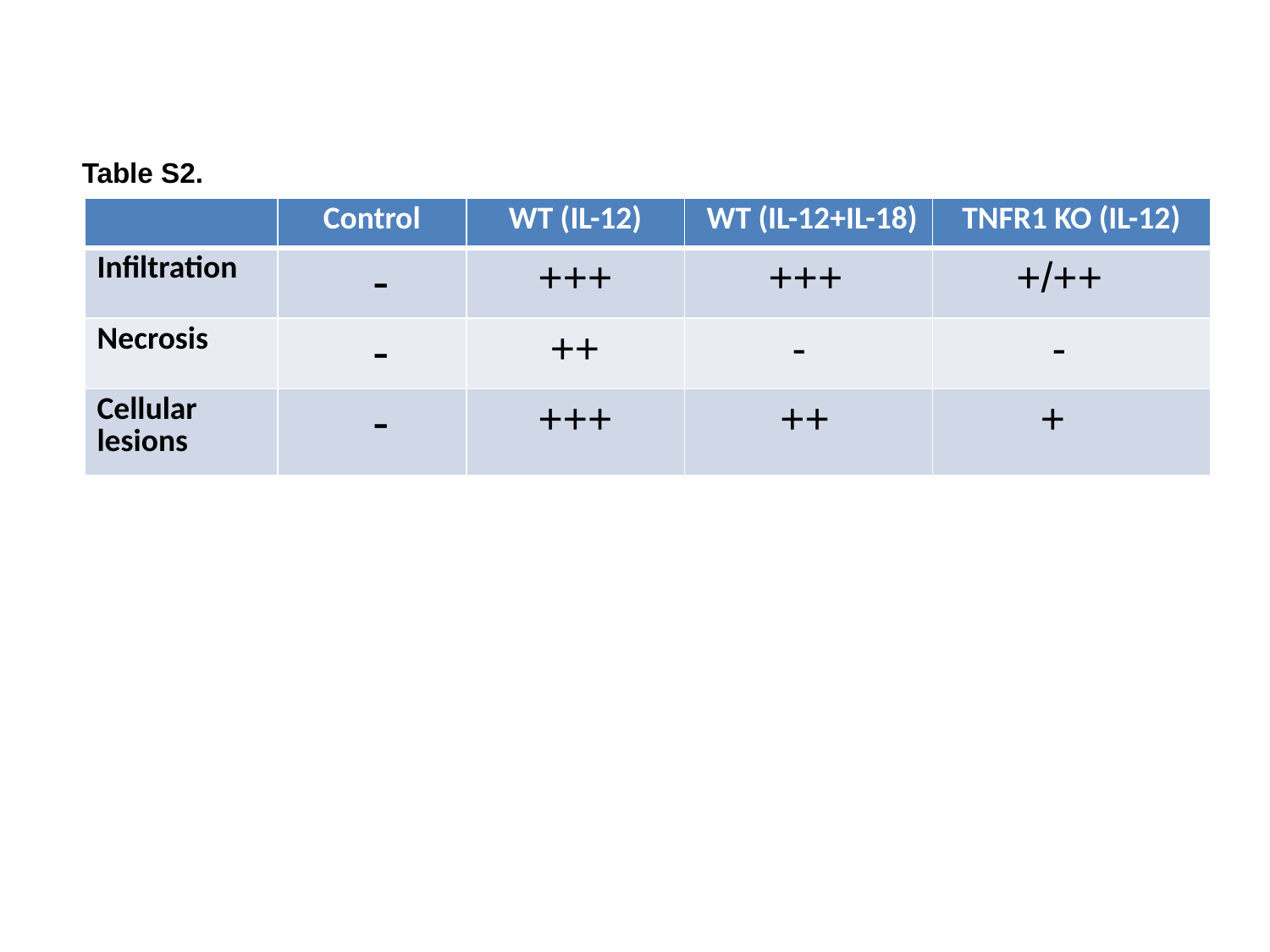

Table S2.
| | Control | WT (IL-12) | WT (IL-12+IL-18) | TNFR1 KO (IL-12) |
| --- | --- | --- | --- | --- |
| Infiltration | - | +++ | +++ | +/++ |
| Necrosis | - | ++ | - | - |
| Cellular lesions | - | +++ | ++ | + |

Supplement: Table S2 — Hepatic pathology in mice treated with systemic IL-12 or IL-12+IL-18. Quantitative analysis of the indicated pathological conditions in B6 or TNFαR1 KO mice treated with control, IL-12 or IL-12+IL-18 cDNAs. Infiltration = mononuclear cells and hematopoeisis. Cellular lesions = Cell balloning and Mallory and Councilman bodies. - = no pathology; + = mild; ++ = moderate; +++ = marked (PPTX) [file pone.0090116.s002.pptx]
